# Factors influencing caregiver burden during perioperative care for children with pediatric central nervous system tumors: an analysis of multidimensional burden and associated determinants

**DOI:** 10.3389/fped.2025.1607200

**Published:** 2025-11-19

**Authors:** Yanjun Yang, Jiahui Fu, Yuwen Fu, Jia Fu

**Affiliations:** 1 Department of Neurosurgery, Peking University International Hospital, Beijing, China; 2 Department of Pediatric, Peking University International Hospital, Beijing, China

**Keywords:** children with central nervous system tumors, perioperative period, care burden, caregivers, contributing factors

## Abstract

**Objectives:**

To investigate the care burden of caregivers of children with central nervous system (CNS) tumors during the perioperative period and analyze the influencing factors of this burden.

**Methods:**

This study enrolled primary caregivers of perioperative children with CNS tumors who underwent neurosurgery at Peking University International Hospital between December 2019 and July 2021. Data were collected via questionnaires (on admission and before discharge) and semi-structured interviews. The Zarit Burden Interview (ZBI-Chinese version) was used to measure care burden. Statistical analyses included the Shapiro–Wilk normality test, paired *t*-test, one-way analysis of variance (ANOVA), and stepwise regression. Semi-structured interview data were analyzed using Colaizzi's seven-step phenomenological approach.

**Results:**

Among the 67 primary caregivers enrolled in this study, the mean care burden score was 29.57 ± 7.22 on admission and 33.90 ± 7.89 before discharge (paired *t* = 4.212, *P* < 0.001), with most scores falling within the mild burden range (20–39 points). Three main factors influencing pre-discharge care burden were identified via stepwise regression: postoperative nutritional disorders (*β* = 0.213, *P* = 0.071, near statistical significance), current employment status of caregivers (*β* = 0.264, *P* = 0.022), and length of hospital stay (*β* = 0.256, *P* = 0.028). Qualitative analysis extracted five themes: navigating the medical system (logistical/access challenges), heavy psychological burden, lack of care knowledge, family-economic pressure, and avoidance of future uncertainty.

**Conclusion:**

Caregivers of the children with pediatric CNS tumours experience significant perioperative care burden, which was higher before discharge than on admission. Interventions targeting postoperative nutritional management, caregiver employment support, and shortened hospital stays (when clinically feasible) may reduce burden. Strengthening caregiver education, psychological support, and social security systems is also recommended.

## 1 Introduction

Data indicate (1) that the incidence of children's cancer is increasing annually worldwide; 30,000–40,000 new cases of childhood cancer are diagnosed in China each year, among which central nervous system (CNS) cancer is the second most common type. Children with cancer require family members to provide caregiving assistance throughout their illness, supporting both their lifestyle and medical needs. Due to the long treatment cycle, the care process requires that caregivers devote a significant amount of time and energy, but they may lack knowledge on how to provide care, have insufficient social support, or find the burden of care to be increasingly heavy. Mental and emotional distress (2–8), such as anxiety, irritability, and sadness can occur; these negative emotions not only affect the caregivers themselves but also indirectly impact (2, 3) the child's treatment and rehabilitation.

Existing studies on caregiver burden in pediatric CNS tumor care have three key limitations. Firstly, overemphasis on child-related factors. Most studies focus on the impact of the child's disease severity or treatment type (9, 10), while neglecting the multi-dimensional interaction between social support (e.g., medical insurance coverage, family assistance), caregivers' own characteristics (e.g., employment status, health literacy), and care burden. Secondly, inadequate exploration of burden dynamics. Few studies compare care burden at different perioperative stages (e.g., admission vs. discharge) or explain why burden may increase before discharge, leaving a gap in understanding the temporal changes of burden. Finally, underdeveloped mechanism research. The formation mechanism of care burden (e.g., how economic pressure mediates the relationship between disease severity and psychological burden) remains unclear, and statistical methods often lack validation for non-normal data or small sample sizes.

This study aimed to fill these gaps by integrating raw data on caregiver characteristics, child clinical conditions, and psychological indicators, providing evidence for personalized interventions.

## 2 Materials and methods

### 2.1 Study participants

Primary caregivers of children with CNS tumors admitted to the Neurosurgery Department of our hospital from December 2019 to May 2021 were included. Inclusion criteria: (1) Children under 14 years of age; (2) Primary caregivers (preferring those that had taken care of the children for a long time, and those still taking care of the child during the perioperative period); (3) Children initially diagnosed with CNS tumors using imaging and expected to be treated with surgery; (4) Caregivers with no critical disease; (5) Caregivers who were able to read, write, and understand Chinese; and (6) Patients voluntarily participated in this study and signed informed consent.

Exclusion criteria: (1) Caregivers with a history of mental illness; (2) Pregnant caregivers; (3) Children with a previous history of other operations within the past six months; (4) Caregivers who dropped out midway or children who died.

This study was approved by the hospital ethics committee [KY202009210005] and conducted in accordance with the Declaration of Helsinki. Informed consent was obtained from all participants, with detailed study information provided to caregivers before enrollment.

### 2.2 Questionnaire surveys

1. General information questionnaire: Collected data on children (age, sex, diagnosis, tumor resection status, postoperative ICU stay, length of hospital stay) and caregivers (age, gender, education level, employment status, medical expense source, disease knowledge).
2. Zarit Burden Interview (ZBI—Chinese version): 22 items, including two dimensions (personal burden, responsibility burden) and one total burden item (not included in dimensions). Scoring: Each item is scored on a 0–4 scale, with a total score ranging from 0 to 88. Classification: 0–19 (no/small burden), 20–39 (mild), 40–59 (moderate), ≥60 (severe). The scale's Cronbach's *α* = 0.87 (11), indicating good reliability and validity.

### 2.3 Data collection methods

Data were collected on the first day of the child's admission and one day before discharge. Incomplete questionnaires were completed by caregivers within 30 min under the guidance of researchers to ensure data integrity.

### 2.4 Semi-structured interviews

Interviews were conducted during the child's hospitalization (30–60 min per interview) to identify potential burden factors for questionnaire design (serving as pre-survey support). The interview outline encompassed perceived medical treatment experiences, disease-related knowledge, the dual impact on family and work, and perceived support needs of the caregivers. A total of 67 caregivers were interviewed (56 females, 11 males; 60 parents, 7 non-immediate relatives).

### 2.5 Statistical analysis

1. **Normality Test:** The Shapiro–Wilk test was used to assess the distribution of Zarit Burden Interview (ZBI) scores. The results showed that the ZBI scores were approximately normally distributed (W = 0.96, *P* = 0.21), which supported the application of parametric tests, including analysis of variance (ANOVA) and regression analysis.
2. **Pairwise Comparison of Repeated Measures**: A paired *t*-test was used to compare ZBI scores upon hospital admission and prior to patient discharge, so as to address the repeated measurement design.
3. **Variable Screening**: For stepwise regression analysis, variables with a *P*-value < 0.1 in univariate analysis (combined with clinical plausibility) were included as candidate variables, aiming to avoid omitting potential influencing factors.
4. **Sensitivity Analysis**: Poisson regression was used to verify the robustness of the stepwise regression results. Consistent trends were observed, which confirmed the reliability of the primary findings.

SPSS 21.0 software was used for all statistical analyses. A *P*-value < 0.05 was considered statistically significant.

### 2.6 Qualitative data analysis

Qualitative data were analyzed using Colaizzi's seven-step phenomenological method, as follows: (1) comprehensive reading of all interview transcripts; (2) extraction of key statements related to the research theme; (3) coding of recurring views and experiences; (4) collation and formation of initial themes; (5) detailed description of core themes; (6) sublimation of theme connotations; (7) respondent verification to ensure the authenticity and representativeness of themes.

## 3 Results

### 3.1 Quantitative study results

#### 3.1.1 General information of children and caregivers

A total of 67 children were included, with a mean age of (6.9 ± 3.8) years (range: 1–14 years). Among them, 47 were males (70.15%) and 20 were females (29.85%); 46 cases underwent complete tumor resection, 59 cases were transferred to the intensive care unit (ICU) postoperatively, and 54 cases developed postoperative nutritional disorders.

For the caregivers, the mean age was (32.2 ± 5.1) years (range: 22–62 years). There were 11 males (16.41%) and 56 females (83.58%); 17.91% (12/67) had an educational level of junior high school or below, 61.19% (41/67) had a senior high school or junior college education, and 20.90% (14/67) held a bachelor's degree or above. Additionally, 79.10% (53/67) had little or no knowledge of the disease; 61.19% (41/67) took temporary leave from work, 16.42% (11/67) resigned, and 22.39% (15/67) were unemployed.

#### 3.1.2 Comparison of caregiver burden scores

Most caregivers presented with mild burden (20–39 points) upon hospital admission (89.55%, 59/67) and prior to patient discharge (73.13%, 49/67) (Table 1). In contrast, the proportion of caregivers with moderate burden (40–59 points) increased from 5.97% (4/67) on admission to 25.37% (17/67) before discharge. No cases of severe burden (≥60 points) were observed. The ZBI scores prior to discharge were significantly higher than those upon admission (Table 2), confirming a dynamic increase in caregiver burden during the perioperative period.

**Table 1:** Comparison of Pre-discharge ZBI scores by caregiver socioeconomic characteristics (*n* = 67).

Variable	Subgroup	*n*	Pre-Discharge ZBI (Mean ± SD)	*F*/*t* value	*P*-value
Age (years)	20–30	16	37.75 ± 9.04	3.591	0.033^*^
	31–40	45	32.16 ± 6.26		
	>40	6	36.50 ± 12.17		
Relationship with child	Immediate Family	60	33.07 ± 6.99	2.626	0.011^*^
	Non-Immediate Family	7	41.00 ± 11.80		
Employment status	Temporary Leave	41	32.29 ± 5.66	5.022	0.009**
	Resigned	11	32.55 ± 8.82		
	Unemployed	15	39.27 ± 10.32		
Medical expense source	Out-of-Pocket	2	36.50 ± 19.09	4.809	0.004**
	Beijing Healthcare	4	32.00 ± 3.91		
	Urban Healthcare	45	31.84 ± 6.68		
	Rural Cooperative	16	39.75 ± 8.02		

One-way ANOVA or independent samples *t*-test was used.

* *P* < 0.05.

** *P* < 0.01.

**Table 2:** Comparison of ZBI total scores on admission and before discharge (*n* = 67).

Item	On Admission (Mean ± SD)	Before Discharge (Mean ± SD)	*t*	*P*-value
ZBI total score	29.57 ± 7.22	33.90 ± 7.89	4.212	<0.001

A paired *t*-test was used for comparison.

#### 3.1.3 Univariate analysis of care burden influencing factors

##### 3.1.3.1 Socioeconomic factors

Caregivers with the following characteristics had significantly higher pre-discharge burden: aged 20–30 years (37.75 ± 9.04), non-immediate family relationship (41.00 ± 11.80), unemployed (39.27 ± 10.32), and out-of-pocket medical expenses (36.50 ± 19.09) (all *P* < 0.05) (Table 3).

**Table 3:** Comparison of Pre-discharge ZBI scores by child's clinical conditions (*n* = 67).

Variable	Subgroup	*n*	Pre-Discharge ZBI (Mean ± SD)	*F*/*t* value	*P*-value
Tumor resection	Complete	46	35.28 ± 7.63	2.189	0.032*
	Incomplete	21	30.86 ± 7.77		
Postoperative ICU stay	Yes	59	34.68 ± 7.77	2.271	0.026*
	No	8	28.13 ± 6.62		
Length of hospital stay	≤7 days	2	27.00 ± 12.72	4.929	0.004**
	8–14 days	15	32.53 ± 5.99		
	15–30 days	42	33.02 ± 7.30		
	>30 days	8	42.75 ± 8.27		
Postoperative nutritional disorders	Yes	54	35.22 ± 7.75	2.964	0.004**
	No	13	28.38 ± 6.06		
Postoperative swallowing difficulty	Yes	4	40.75 ± 9.63	1.822	0.073
	No	63	33.46 ± 7.65		
Postoperative fever	Yes	61	34.44 ± 7.94	1.841	0.070
	No	6	28.33 ± 5.00		

One-way ANOVA or independent samples *t*-test was used.

* *P* < 0.05.

** *P* < 0.01.

##### 3.1.3.2 Child condition factors

Children with incomplete tumor resection, no postoperative ICU stay, shorter hospital stay (≤7 days), or no postoperative nutritional disorders were associated with lower caregiver burden (all *P* < 0.05). Variables with *P* ≈ 0.07 (postoperative swallowing difficulty: 40.75 ± 9.63 vs. 33.46 ± 7.65, *P* = 0.073; postoperative fever: 34.44 ± 7.94 vs. 28.33 ± 5.00, *P* = 0.070) were near statistical significance but not included in regression due to small sample sizes (Table 4).

**Table 4:** Stepwise regression analysis of Pre-discharge care burden influencing factors (*n* = 67).

Independent variable	Beta	SE	Standardized beta	*t*	*P*-value
Constant term	26.276	5.72	–	4.594	<0.001
Postoperative nutritional disorders	4.226	2.298	0.213	1.839	0.071
Caregiver employment status	2.496	1.06	0.264	2.355	0.022*
Length of hospital stay	3.042	1.348	0.256	2.256	0.028*

Adjusted R^2^ = 0.215, F = 7.022, *P* < 0.001.

* *P* < 0.05.

#### 3.1.4 Stepwise regression analysis of Pre-discharge care burden

Three factors explained 21.5% of the variance in pre-discharge burden (adjusted R^2^ = 0.215): (1) postoperative nutritional disorders (*β* = 0.213, *P* = 0.071, near significance); (2) caregiver employment status (*β* = 0.264, *P* = 0.022); (3) length of hospital stay (*β* = 0.256, *P* = 0.028) (Table 4).

#### 3.1.5 Qualitative research results

Semi-structured interviews revealed five core themes, which were derived from the shared experiences of caregivers:

##### 3.1.5.1 Navigating the medical system: logistical and access challenges

Caregivers encountered substantial barriers when seeking specialized care. Many reported traveling across multiple regions—from county- and municipal-level hospitals to provincial institutions, and ultimately to hospitals in Beijing—primarily due to limited local expertise in the diagnosis and treatment of pediatric CNS tumors. During this process, confusion regarding specialist appointment registration (e.g., relying on roommates or online resources to secure appointments) further exacerbated their stress. The COVID-19 pandemic introduced additional hurdles, such as transportation restrictions, stringent nucleic acid testing requirements for accommodation, and policies limiting caregiver accompaniment in some hospitals—all of which further depleted caregivers' energy.

##### 3.1.5.2 Severe psychological burden

Caregivers expressed intense negative emotions, including guilt and anxiety. A common sentiment was self-blame, with caregivers stating, “I didn't take good care of my son—he's so young and has to suffer so much”. Anxiety focused on surgical risks (e.g., impacts on intelligence or mobility) and long-term uncertainty; some caregivers delayed informing grandparents about the diagnosis to avoid causing additional distress. Postoperative recovery fluctuations (e.g., fever or slow rehabilitation) further heightened anxiety.

##### 3.1.5.3 Insufficient knowledge of the disease and care practices

Caregivers had limited understanding of pediatric CNS tumors. Owing to the lack of expertise among local doctors, they often relied on inconsistent online information or experiences shared by other caregivers. Key knowledge gaps during the perioperative period included: surgical procedures, preoperative skin preparation (e.g., discussing hair shaving with appearance-sensitive children), and postoperative care (e.g., recognizing signs of discomfort or managing pain).

##### 3.1.5.4 Family and financial pressures

The child's illness disrupted both family life and financial stability. Many caregivers took temporary leave or resigned from their jobs, with one explaining, “I used up all my leave days and had to quit my job, while my husband also needs to take leave frequently”. For most families, medical expenses accounted for over 50% of their annual income, and some resorted to borrowing money to cover treatment costs. Caregivers with other children also experienced guilt over divided attention, as siblings were often left in the care of grandparents.

##### 3.1.5.5 Avoidance of future uncertainty

To cope with anxiety, caregivers prioritized immediate surgical outcomes over long-term planning. Comments such as, “I dare not think about the future—I just need to get through the surgery first”, reflected their strategy of focusing on short-term tasks to manage stress. Many also acknowledged, “Life will never be the same again”, which underscored a sense of loss regarding the life circumstances of the child after illness.

## 4 Discussion

### 4.1 Postoperative malnutrition and length of hospital stay

Surgical trauma places children in a state of stress, leading to slowed gastrointestinal peristalsis and decreased appetite. Additionally, surgery may damage nerves or muscles involved in swallowing, causing dysphagia and thereby impairing food intake (12). In this study, both postoperative malnutrition and length of hospital stay were identified as key factors influencing caregiver burden. Malnourished children require specialized dietary planning and monitoring, increasing caregivers' daily tasks and psychological stress. During the pandemic, limited access to palatable meals further complicated nutrition management. Postoperative malnutrition is characterized by acute nutritional imbalance, which can be rapidly and effectively corrected with early nutritional support. Therefore, providing caregivers with systematic education on managing nutritional disorders, neurological symptoms (e.g., lethargy, seizures), and other complications as well as rehabilitation—such as developing age-appropriate visual educational materials (e.g., illustrated postoperative care manuals) and deploying artificial intelligence (AI)-supported rehabilitation guidance tools—helps reduce caregiver burden and alleviate anxiety caused by “fear of missing warning signs and harming the child due to lack of experience”.

Furthermore, due to cumulative physical and mental exhaustion, caregivers exhibited a significantly higher burden score before discharge compared to that at admission. Postoperative care (e.g., nighttime fever management, wound monitoring) was more demanding than preoperative examination support, which leading to sleep deprivation and emotional fatigue.

Longer stays increased care duration and indirect costs (e.g., accommodation for caregivers during the pandemic). In this study, some children experienced prolonged hospital stays due to complications such as malnutrition and fever, requiring caregivers to invest more time and effort. Additionally, pandemic-related travel restrictions and other limitations further exacerbated this challenge. For non-only-child families, additional expenses for sibling care (e.g., hiring help) further amplified burden.

### 4.2 Caregiver employment status

In this study, caregiver employment status was identified as one of the key factors influencing caregiver burden. Additionally, analysis of qualitative research findings revealed that the impact of caregiver employment status on caregiver burden manifests in five dimensions: pressure from direct medical expenditures, accumulation of non-medical costs, limited treatment decision-making, increased psychological burden, and concerns regarding long-term prognosis.

Specifically, 38.81% of caregivers in this study were resigned or unemployed, while 61.19% were on temporary leave. Financial pressure affected both the selection of treatment plans and investment in the child's post-treatment rehabilitation. Notably, only 7.46% of the children in this study had purchased commercial insurance, meaning most families had to bear high out-of-pocket medical expenses. Although specific subsidies were available for application in some regions, the application process was cumbersome. This financial burden not only exacerbated caregivers' psychological distress but also reduced their ability to provide continuous, high-quality care—consistent with the conclusion from previous research that financial stress exacerbated psychological burden and reduced caregiving efficiency (13).

Therefore, it is recommended that governments strengthen the development of medical alliances to improve the level of pediatric oncology diagnosis and treatment services in underdeveloped areas, thereby reducing the burden of cross-regional medical care for families (14). Furthermore, it is suggested to expand the reimbursement coverage for major diseases involving pediatric central nervous system tumors and simplify claim settlement procedures to alleviate financial barriers related to medical costs.

For caregivers themselves, taking long-term leave or resigning had a significant impact on their own career development and social status. It is thus recommended that local communities provide flexible part-time work opportunities for caregivers and establish community-level rehabilitation programs to offer supplementary support for the employment of unemployed caregivers.

### 4.3 Caregivers' psychological Status

Caregivers' psychological status changes with different stages of the child's illness. Specifically. This study showed that preoperatively, caregivers expressed expectations for the early scheduling of surgery and positive postoperative outcomes; postoperatively, they worried about their ability to manage various care-related issues. Before discharge, caregivers exhibited significant uncertainty regarding their own ability to provide home-based care. They worried about home care competence (15) (e.g., “Can I manage rehabilitation alone?”), and mothers—who accounted for 77.6% of caregivers—experienced higher illness uncertainty than fathers (16, 17), further amplifying anxiety.

This study revealed Caregivers of children with complete resection had higher burden (Table 3), which may be associated with unmet expectations and a shift in focus. Caregivers of children with incomplete resection expected subsequent adjuvant treatments (18) (e.g., radiotherapy) and viewed this as “controllable” reducing anxiety. In contrast, caregivers of children with complete resection focused on long-term neurological prognosis (e.g., cognitive or mobility impacts), and minor recovery delays (e.g., slow gait improvement) exacerbated fear of “permanent sequelae”.

Therefore, if healthcare providers offer one-on-one psychological counseling services to families in need—helping caregivers address suppressed emotions (many caregivers hide their exhaustion to appear “strong” in front of their children)—it can help alleviate caregivers' psychological stress. Additionally, establishing peer support groups to reduce loneliness through experience sharing and prevent overly high expectations of surgical outcomes can help normalize stress.

Setting up dedicated WeChat groups for post-discharge follow-up to enable real-time communication between caregivers and the medical team is currently a relatively direct communication method. However, a conflict exists between healthcare providers' regular working hours and the time required to respond to messages in these WeChat groups. Furthermore, due to the limitation of non-face-to-face communication, there are certain limitations in the accuracy of caregivers' observations and descriptions of their children's conditions—these are key barriers to communication via WeChat groups.

## 5 Study limitations

1. No follow-up data on post-discharge burden were collected; future studies should track burden for 3–6 months after discharge.
2. The sample size (*n* = 67) was small due to strict inclusion criteria; larger multi-center studies are needed to validate results.

## 6 Summary

This study dynamically assessed perioperative care burden of caregivers of children with pediatric CNS tumours, confirming higher burden before discharge than on admission. Key influencing factors included postoperative nutritional disorders, caregiver employment status, and length of hospital stay. By integrating qualitative and quantitative methods, this study provides a foundation for developing targeted interventions to reduce caregiver burden and improve child care quality.

## Data Availability

The raw data supporting the conclusions of this article will be made available by the authors, without undue reservation.
